# Effects of Storage Temperature and Time on Stability of Serum Tacrolimus and Cyclosporine A Levels in Whole Blood by LC-MS/MS

**DOI:** 10.1155/2015/956389

**Published:** 2015-04-09

**Authors:** İbrahim Kaplan, Hatice Yüksel, Osman Evliyaoğlu, M. Kemal Basarali, Gülten Toprak, Leyla Çolpan, Velat Şen

**Affiliations:** ^1^Department of Biochemistry, Faculty of Medicine, Dicle University, Diyarbakir, Turkey; ^2^Department of Biochemistry, Okmeydani Hospital, Istanbul, Turkey; ^3^Department of Pediatrics, Faculty of Medicine, Dicle University, Diyarbakir, Turkey

## Abstract

Tacrolimus and cyclosporine A are immunosuppressant drugs with narrow therapeutic windows. The aim of this study was to investigate the stability of tacrolimus and cyclosporin A levels in whole blood samples under different storage conditions. Whole blood samples were obtained from 15 patients receiving tacrolimus and 15 patients receiving cyclosporine A. Samples were immediately analyzed and then stored at different conditions (room temperature (24°C−26°C) for 24 hours, +4°C for 24 and 48 hours, and −20°C for one month) and then analyzed again. For tacrolimus, there was a significant difference between samples analyzed immediately and those kept 24 hours at room temperature (*P* = 0.005) (percent change 32.89%). However, there were no significant differences between the other groups. For cyclosporine A, there was a significant difference between samples analyzed immediately and those kept 24 hours (*P* = 0.003) (percent change 19.47%) and 48 hours (*P* = 0.002) (percent change 15.38%) at +4°C and those kept 24 hours at room temperature (*P* = 0.011) (percent change 9.71%). Samples of tacrolimus should be analyzed immediately or stored at either +4°C or −20°C, while samples of cyclosporine A should be analyzed immediately or stored at −20°C.

## 1. Introduction

Preanalytical problems are the major source of laboratory errors, accounting for 60–70% of total errors encountered within the total testing process. Preanalytical errors not only may lead to spurious test results, but also, more importantly, may influence patient care, even dramatically [1].

The immunosuppressive drugs tacrolimus (TAC) and cyclosporine A (CsA) are used in organ transplantation [2]. These drugs are calcineurin inhibitors that block interleukin-2 (IL-2) transcription and T lymphocyte signal transduction. They are used for prophylaxis against tissue rejection after liver, heart, and kidney transplantation. In addition, CsA is used in the treatment of graft-versus-host reactions, in severe psoriasis, and after bone marrow transplantation [3].

TAC is a lipophilic drug that has variable absorption and is extensively metabolized in the liver [4, 5]. Approximately 95% of TAC is eliminated by the biliary route, while 5% is excreted unchanged in the urine [6]. Cytochrome P-450 3A4 and 3A5 metabolize this drug in the small intestine and liver [7]. CsA is a substrate of the P-glycoprotein (ABCB1) efflux transporter and primarily metabolized by CYP3A4 and CYP3A5. It is nephrotoxic and causes damage to the kidney vasculature, glomerulus, and proximal tubule at high dose. CsA is thought to induce oxidative stress at the mitochondrial level [8].

CsA and TAC have narrow therapeutic ranges. CsA and TAC cause numerous side effects, including neurological, hepatic, renal, and immunological complications. Dose adjustment or discontinuation may be necessary, because these complications occur in a significant number of patients [9, 10].

A significant consequence of overdosage of CsA and TAC is nephrotoxicity, while underdosage of these drugs can result in transplant rejection [11]. Therefore, in order to reduce the risk of organ rejection and toxicity, therapeutic drug monitoring plays a key role in maintaining therapeutic blood levels of these drugs [12].

Immunoassays methods (such as microparticle enzyme immunoassay and cloned enzyme donor immunoassay) and liquid chromatography based methods (such as high-performance liquid chromatography (HPLC) with ultraviolet detection, LC-mass spectrometry (LC-MS), and LC-tandem mass spectrometry (LC-MS/MS)) are used for determination of concentrations of immunosuppressive drugs. Most routine laboratories use immunological methods. In immunological techniques, overestimation of concentrations is a major problem because cross reactions can occur with some metabolites. The LC-MS/MS method is more sensitive and specific than immunological methods and has significant advantages over other methods. Therefore this method is considered to be the gold standard in therapeutic drug monitoring [13–15].

These drug levels are not analyzed in every hospital laboratory, but rather the samples are transferred to laboratories in other cities. Therefore samples cannot be analyzed immediately. It is known that prolonged storage time and inappropriate temperature may cause incorrect results for many analyses. In this study, we investigated the effect of storage time and temperature on drug concentrations in whole blood samples in order to determine the optimal storage conditions for accurate measurement of these drugs.

## 2. Materials and Methods

### 2.1. Materials

The research protocol was approved by the Local Ethics Committee of Dicle University School of Medicine. Blood samples from 15 patients using TAC and 15 patients using CsA were included in the study. Nine patients using TAC had received kidney transplants, and the 6 patients had received liver transplants. All the patients using CsA were patients who had received kidney transplants. Patients using any other drug were excluded from the study.

### 2.2. Blood Sampling

Blood samples (2 mL) were drawn into K_2_EDTA tubes (Becton Dickinson Company, USA) from the subjects in the morning after an overnight fast and were transferred to laboratory within 1 minute.

All samples were divided into three aliquots. One aliquot was stored at −20°C for one month and then analyzed. The second aliquot was immediately analyzed and kept for 24 hours at room temperature (24°C–26°C) and measured again. The third aliquot was kept at +4°C and analyzed 24 and 48 hours later. Immediately analyzed samples were considered as the baseline value. The drug levels were measured by LC-MS/MS method.

### 2.3. Sample Preparation

TAC and CsA standard stock solutions in liquid form were purchased from Cerilliant Inc. (Round Rock, Texas) and stored at +4°C. 0.1 M ZnSO_4_ precipitation solution containing the internal standards cyclosporin D (1234/1217) and ascomycin (809.6/756.6) was prepared before sample preparation. Whole blood samples were put into a tube as 100 *μ*L volume and 300 *μ*L precipitation reagent was added. Samples were immediately vortexed 30 seconds and left 5 minutes at the room temperature. After vortexing for additional 5 sec, the tubes were centrifuged for 10 minutes at 10000 rpm at 4°C. The supernatant was transferred to a clean vial for quantitation. Three level quality control serums (QCs) were purchased from UTAK (Valencia, CA).

### 2.4. LC-MS/MS Conditions

The LC-MS/MS was performed using an IONICS 3Q 120 triple quadrupole mass spectrometer (Bolton, ON Canada) with a Shimadzu Ultra-Fast Liquid Chromatograph (UFLC) system. 20 *μ*L of supernatant was loaded on a porous R1/20 pretreatment column (30 × 2.1 mm) for on-line washing with water for 0.25 minutes at a liquid flow rate of 3 mL/min and then eluted by an Imtakt Cadenza CD-C18HT analytical column (50 × 2.0 mm, 3 *μ*m) at flow rate of 0.6 mL/min using Solvent A (water : methanol = 98 : 2, v/v, with 0.1% formic acid and 10 mM ammonium acetate) and Solvent B (water : methanol = 2 : 98, v/v, with 0.1% formic acid and 10 mM ammonium acetate).

The sensitivity of TAC and CsA was 0.1 ng/mL and 1.0 ng/mL respectively. And CV% of TAC and CsA was 5.92% and 5.08% respectively. Recovery of the method was >98%.

### 2.5. Statistical Analysis

SPSS for Windows 15.0 statistical program was used for statistical analysis. Repeated measurements made from the same samples kept at different storage conditions were tested using the Wilcoxon test. A *P* value of less than 0.05 was considered to be significant.

## 3. Results

The results of TAC and CsA levels are shown in Table 1.

**Figure 1:**
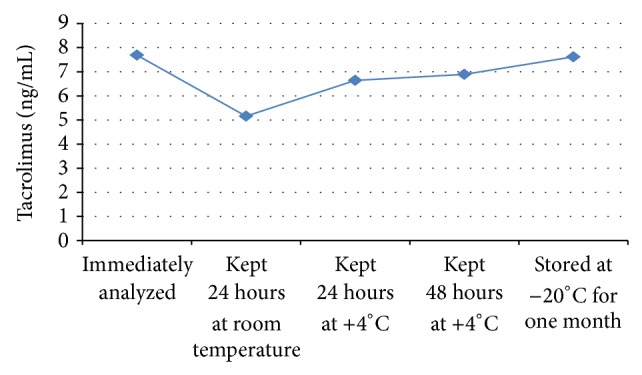
Tacrolimus levels of the samples stored under different storage conditions.

*Tacrolimus*. We did not find a significant difference between samples analyzed immediately and those kept at +4°C for 24 hours (*P* = 0.241) (percent change −13.78%) and 48 hours (*P* = 0.285) (percent change −10.4%), and there was no significant difference between samples analyzed immediately and those stored at −20°C for one month (*P* = 0.646) (percent change −0.91%). However, there was a significant difference between samples analyzed immediately and those kept at room temperature for 24 hours (*P* = 0.005) (percent change −32.89%) (Figure 1).

**Figure 2:**
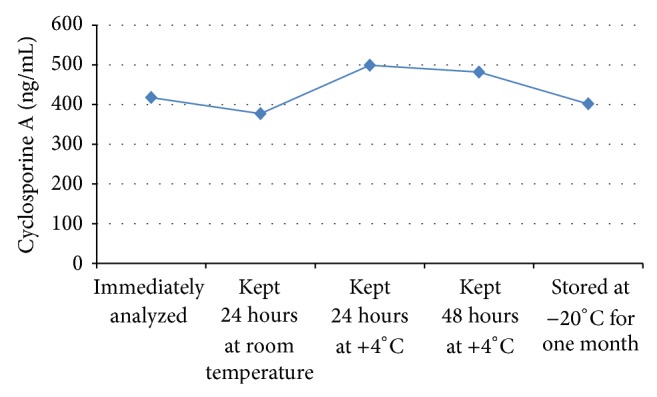
Cyclosporine A levels of the samples stored under different storage conditions.

*Cyclosporine A*. We found significant differences between CsA samples that were immediately analyzed and those stored under different conditions (Figure 2). The results of the samples that were kept at +4°C for 24 hours (*P* = 0.003) (percent change 19.47%) and 48 hours (*P* = 0.002) (percent change 15.38%) and those kept at room temperature (*P* = 0.011) (percent change −9.71%) for 24 hours were significantly different. However, there was no significant difference between samples analyzed immediately and samples that were stored at −20°C for one month (*P* = 0.173) (percent change −3.82%).

## 4. Discussion

In this study we evaluated the effects of different storage conditions on the detected concentrations of TAC and CsA that were measured in the same samples.

Our results showed that the stability of TAC was affected by storage at room temperature for 24 hours (*P* = 0.005), but the samples were stable if they were stored at +4°C for one day or two days, or at −20°C for 1 month (*P* = 0.241, *P* = 0.285, and *P* = 0.646, resp.). However, CsA levels were decreased in the samples that were kept at room temperature for one day or two days and in the samples that were kept at +4°C for 1 day (*P* = 0.003,  *P* = 0.002, and  *P* = 0.011, resp.) They were stable only at −20°C (*P* = 0.173). Therefore, the drug levels of samples that are left at room temperature and not stored properly will be inaccurate.

Organ transplant recipients must take immunosuppressant drugs to prevent rejection. To maximize drug efficacy and minimize adverse events, accurate measurements of blood immunosuppressant concentrations and dose adjustments are important [16].

Therapeutic drug monitoring must deliver a sensitive and accurate measurement of an administered drug to be a successful diagnostic instrument in patient care [17]. Therefore, the optimization of sample processing especially the run-time and storage conditions in the preanalytical period is important for the accuracy of measurements.

Sample stability studies for various analytes were made previously by some researchers. Taylor and Sethi evaluated 27 biochemical analytes in their study and determined that some results of biochemical parameters changed in different conditions and storage time [18]. Gao et al. reported that tumor markers (AFP, CEA, and CA125) are significantly affected by long-term frozen storage [19]. However in another study repeated freezing to −70°C and thawing had no meaningful effects at the plasma and serum concentrations of hormones [20].

Studies for stability of immunosuppressant drugs were made previously by some researchers. Ingels et al. in their study stored 6 whole blood samples at room temperature, +4°C and −20°C for one week, and they observed that there was no change in the levels of TAC. They used microparticle enzyme immunoassay technology for TAC measurement [21]. In another study Smith and Sephel researched the stability of CsA. They tested 10 different samples from eight patients. Specimens were analyzed on the day of collection and repeatedly for nine to 13 days after storage at room temperature (20–24°C) and 37°C with radioimmunoassay method and they observed no significant difference at the results [22].

Annesley et al. in their study using 21 TAC and 13 CsA specimens stored the samples at ambient temperature and +4°C for 7 days and reported that there was no decrease in CsA concentrations over a seven-day period for specimens stored at either ambient temperature or +4°C, while the concentrations of TAC in whole blood stored at ambient temperature for seven days had a slight downward trend (a mean decrease of 5%). The methods were Fluorescence Polarization Immunoassay (FPIA) for CsA and monoclonal antibody microparticle enzyme immunoassay for TAC [23].

Freeman et al. observed that concentrations of TAC were stable at room temperature, +4°C, and −70°C for the two-week study period in their study using the microparticle enzyme immunoassay method. They also reported that the samples stored at −70°C for almost one year and then reanalyzed were found to be stable [24].

In our study, we observed that the stability of TAC did not change over a two-day period for samples stored at +4°C or a one month period at −20°C (Table 1). This was consistent with previous studies. However, concentrations of TAC were decreased in samples that were left out at room temperature. CsA levels were stable at −20°C over a one month period. They were unstable when they were kept at room temperature or +4°C (Table 1). This was not consistent with previous studies. This incompatibility may be caused by the method that we used is different. We used LC-MS/MS in our study because it is a rapid, specific, sensitive, and accurate reference method [25]. Studies showed that LC-MS/MS had significant reproducibility and accuracy advantages compared to Fluorescence Polarization Immunoassay, microparticle enzyme immunoassay, and conventional HPLC-UV methods for the quantitation of TAC and CsA concentrations in whole blood [26, 27]. Based on these results, to accurately determine the level of CsA samples should be measured immediately or stored at −20°C until analysis.

In conclusion, the stability of TAC and CsA depends on storage conditions, which must be considered for accurate measurement. Furthermore, these findings can give an idea about the conditions under which the serum samples should be kept for TAC and CsA measurement. Further studies with larger samples are needed to delineate this relationship.

## Figures and Tables

**Table 1:** The cyclosporine A and tacrolimus levels of the samples stored under different storage conditions.

Sample	Cyclosporine A (mean ± SD) (ng/mL) (*n* = 15)	Percent change (%)	Tacrolimus (mean ± SD) (ng/mL) (*n* = 15)	Percent change (%)
Immediately analyzed (baseline value)	417.66 ± 449.02^a,b,c^		7.69 ± 2.62^a^	
Kept 24 hours at room temperature	377.09 ± 398.69	−9.71	5.16 ± 1.84	−32.89
Kept 24 hours at +4°C	499.02 ± 520.05	19.47	6.63 ± 2.29	−13.78
Kept 48 hours at +4°C	481.90 ± 485.28	15.38	6.89 ± 2.95	−10.4
Stored at −20°C for one month	401.67 ± 421.58	−3.82	7.62 ± 4.45	−0.91

^a^ *P* = 0.005 for tacrolimus and *P* = 0.011 for cyclosporine A when compared with the samples kept 24 hours at room temperature.

^b^ *P* = 0.003 when compared with samples kept 24 hours at +4°C.

^c^ *P* = 0.003 when compared with samples kept 48 hours at +4°C.
